# Pretreatment of human retinal pigment epithelial cells with sterculic acid forestalls fenretinide-induced apoptosis

**DOI:** 10.1038/s41598-022-26383-9

**Published:** 2022-12-23

**Authors:** Samuel William, Todd Duncan, T. Michael Redmond

**Affiliations:** Laboratory of Retinal Cell and Molecular Biology, Bldg. 6/Room 112A, National Eye Institute, National Institutes of Health, 6 Center Drive, Bethesda, MD 20892-0608 USA

**Keywords:** Biochemistry, Apoptosis

## Abstract

The ratio of saturated to monounsaturated fatty acids, thought to play a critical role in many cellular functions, is regulated by stearoyl-CoA desaturase (SCD), a rate-limiting enzyme in the biosynthesis of monounsaturated fatty acids. Previously, we observed a decrease in both SCD protein and enzymatic activity in apoptosis induced by fenretinide, a synthetic analog of retinoic acid, in the human retinal pigment epithelial (RPE) cell line ARPE-19. Here, we investigated the effect of pretreating ARPE-19 with sterculic acid, a cyclopropenoic fatty acid inhibitor of SCD, on preventing fenretinide-induced apoptosis, given the role of SCD in cell proliferation and apoptosis. We show that sterculic acid pretreatment prevents the effects of fenretinide-induced apoptosis shown by changes in cell morphology, viability, and caspase-3 activation. Analysis of endoplasmic reticulum (ER)-associated proteins shows that sterculic acid pretreatment reduced the fenretinide-induced upregulation of heme oxygenase-1, ATF3 and GADD153 expression that are in response to reactive oxygen species (ROS) generation. Sterculic acid is as effective as allopurinol in inhibition of xanthine oxidase (XDH), and this may play a role in reducing the potential role of XDH in fenretinide-induced ROS generation. Sterculic acid pretreatment also results in a reduction in SOD2 mRNA expression. Dihydroceramide accumulation, compared to ceramide, and ROS generation indicate that a ceramide-independent pathway mediates fenretinide-induced apoptosis, and ROS mediation is borne out by activation of the NF-κBp50 and NF-κBp65 downstream signaling cascade. Its prevention by sterculic acid pretreatment further indicates the latter’s antioxidant/anti-inflammatory effect. Taken together, our results suggest that sterculic acid pretreatment can mitigate ROS-mediated fenretinide-induced apoptosis. Thus, sterculic acid may serve as a potential antioxidant and therapeutic agent. These effects may be independent of its effects on SCD activity.

## Introduction

Retinal pigment epithelium (RPE) is a polarized monolayer of highly differentiated epithelial cells located between the light-sensing photoreceptor cells and choriocapillaris in the eye. Apart from forming the blood-retinal barrier, RPE participates in the continuous renewal of photoreceptor outer segments (POS), critical for vision. Shed POS contain roughly equal amounts of saturated (including palmitate) and unsaturated fatty acids^1^. This provides RPE with substrates for mitochondrial and peroxisomal fatty acid β-oxidation^2^. Changes in fatty acid metabolism, in particular of saturated fatty acids, are characteristic of many age-related human diseases^3^, as palmitate promotes ceramide accumulation, central in regulating cell fate under different pathological conditions, while decreased palmitate-driven ceramide synthesis curbs insulin resistance^4^.

Desaturation of long-chain saturated fatty acids to monounsaturated fatty acids is catalyzed by stearoyl-CoA desaturase (SCD, SCD1), an endoplasmic reticulum (ER) resident enzyme^5,6^. The saturated:unsaturated fatty acid ratio regulates many membrane properties including structural integrity and fluidity and modulates cell adhesion, migration, and differentiation^7,8^. SCD plays a crucial role in regulating many adverse effects of saturated fatty acids on cell function such as mitochondrial dysfunction and promotion of apoptosis^9^, unfolded protein response pathways^10^, and may play a pivotal role in many age-related diseases including cardiovascular disease, cancer, and type-2 diabetes^3,11,12^. Conversely, oleate and palmitoleate, either by exogenous addition or by endogenous desaturation by SCD, may exert protective effects^13^. Genetic or pharmacological manipulation of SCD have been shown to impair de novo lipid synthesis, decrease cell proliferation, and increase apoptosis in cancer cells^14,15^. Certain fatty acid derivatives are reported to exert antineoplastic activity by inhibiting SCD. Among these, the cyclopropenoic fatty acid sterculic acid is a naturally occurring inhibitor of SCD activity found in the seeds of *Sterculia foetida*. Sterculic acid has been suggested for the treatment of metabolic syndrome, Alzheimer’s disease, cancer, and retinal disorders^16–19^.

Previously, we showed that SCD is expressed in RPE and that its expression is regulated by all-*trans* retinoic acid (atRA) and TGF-β^20,21^. We found that fenretinide (N-(**4**-**h**ydroxy**p**henyl)-**r**etinamide, 4HPR), a synthetic analog of atRA, induces apoptosis in the ARPE-19 human RPE cell line. Fenretinide induces its anti-proliferative and pro-apoptotic effects through retinoic acid receptor (RAR)-dependent or independent mechanisms in several cell lines^22,23^, via production of ROS and ceramide accumulation^24,25^. Earlier, we found that fenretinide-induced apoptosis in ARPE-19 cells occurs via ROS generation and was effectively blocked by RAR antagonists and the antioxidant pyrrolidine dithiocarbamate^23^, and further that apoptosis is mediated via the ubiquitin-dependent proteasomal pathway in response to ER stress along with decrease in SCD protein and enzymatic activity^26^. The present work examines whether pretreatment of cells with sterculic acid can prevent fenretinide-induced ROS generation/apoptosis in ARPE-19 cells. We show that to be the case and dissect the mechanism of this effect.

## Results

### Sterculic acid pretreatment blocks fenretinide-induced apoptosis in ARPE-19 cells

**Figure 1:**
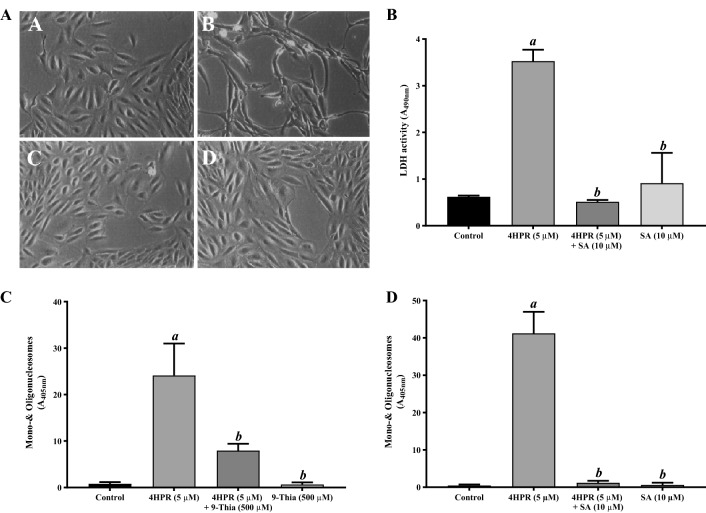
Fenretinide-induced cell death of human ARPE-19 cells is prevented by pretreatment with sterculic acid (SA), an inhibitor of SCD. *Panel A*, phase-contrast micrographs of ARPE-19 cells treated with 5 μM fenretinide (4HPR) in the presence or absence of SA for 24 h. (**A)** Subconfluent cells 2 days after plating. (**B**) Cells treated with 4HPR for 24 h. (**C)** Cells treated with 4HPR in the presence of SA. (**D**) Cells treated with SA alone. Magnification ×100. *Panel B,* Inhibition of fenretinide-induced LDH release from ARPE-19 cells by SA. *Panel C*, Inhibition of fenretinide-induced apoptosis by SCD inhibitor 9-Thia-stearate. *Panel D,* inhibition of fenretinide-induced apoptosis by SA. ARPE-19 cells were treated with 5 μM fenretinide in the presence or absence of the indicated SCD inhibitors for 24 h, then analyzed by apoptosis ELISA as described under “Experimental Procedures”. The values are mean ± SD of three independent experiments performed in quadruplicate. To test for significant differences between treatment groups, one-way analysis of variance (ANOVA) was used. In the graphs ‘*a’* indicates statistical significance difference between fenretinide treated and untreated control, and ‘*b’* indicates statistical significance difference between fenretinide and fenretinide treated with inhibitors, and inhibitors alone (^*a*,*b*^p < 0.001).

The effect of the SCD inhibitor sterculic acid on the human RPE cell line ARPE-19 was investigated. We have shown earlier that fenretinide-induced apoptosis in ARPE-19 cells involves a decrease in SCD protein along with a corresponding decrease in SCD enzyme activity^26^. Therefore, ARPE-19 cells pretreated in the presence or absence of 10 μM sterculic acid were incubated with 5 μM fenretinide for 24 h and their viability assessed. Cells incubated with 5 μM fenretinide appeared to shrink compared to control-treated cells (Fig. 1A, panel A), detached from the bottom of the dish, and began to float in the culture medium (Fig. 1A, panel B), similar to our earlier observations. However, pretreatment of cells with sterculic acid significantly inhibited fenretinide-induced cytotoxicity (Fig. 1A, panel C). The cells pretreated with sterculic acid retained their normal shape and appeared viable by phase contrast microscopy (Fig. 1A, panel D). ARPE-19 cell viability was measured by the release of the cytoplasmic enzyme LDH into the medium as an index of cell death^27^. A significant increase in LDH release was observed after 24 h incubation with 5 μM fenretinide compared to control, in line with light microscopy observations. We found that sterculic acid pretreatment prevented fenretinide-induced LDH release, indicating the involvement of SCD in fenretinide-induced cytotoxicity (Fig. 1B).

To determine whether the reduced ARPE-19 cell survival after treatment with 5 μM fenretinide occurs via apoptosis, we performed apoptosis ELISA, a technique that estimates the amount of cytoplasmic histone-associated DNA fragments that accumulate in cells during apoptosis. As shown in Fig. 1C, fenretinide treatment resulted in generation of DNA fragments, a hallmark of apoptosis. Incubation with 5 μM fenretinide caused a more than tenfold increase in DNA mono/oligonucleosomes over the control at 24 h. Further, the involvement of SCD in fenretinide-induced apoptosis was analyzed in the presence or absence of SCD inhibitors 9-Thia and sterculic acid. A moderate decrease in fenretinide-induced apoptosis was observed in ARPE-19 cells pretreated with 500 μM 9-Thia (Fig. 1C). On the other hand, fenretinide-induced apoptosis was completely blocked by pretreatment with 10 μM sterculic acid (Fig. 1D), as indicated by the levels of mono/oligonucleosomes. Apoptosis was not detected in control cells treated with 9-Thia and sterculic acid alone.

### Fenretinide-induced apoptosis in ARPE-19 cells is mediated by ER stress and this is mitigated by pretreatment with sterculic acid

**Figure 2:**
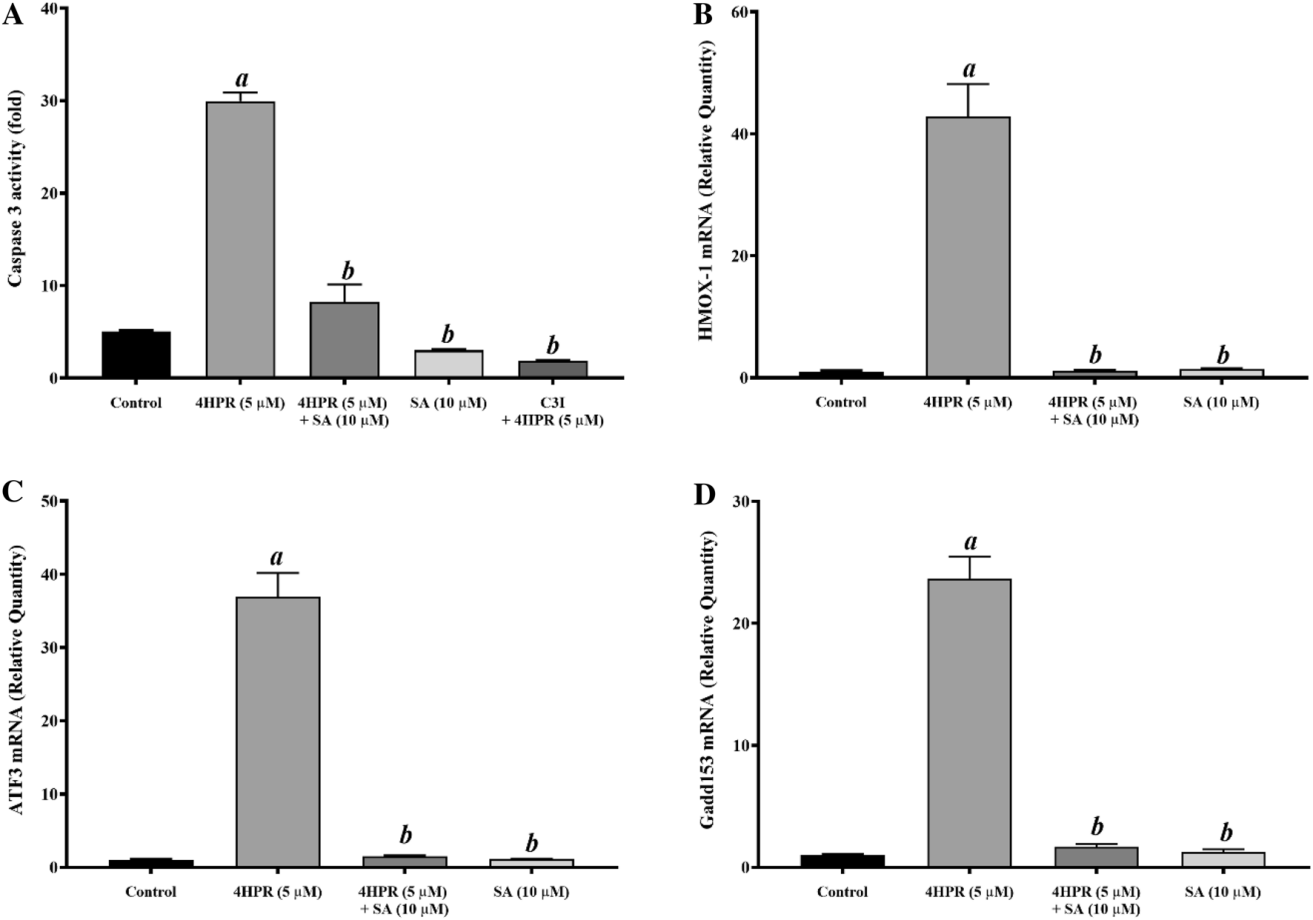
Inhibition of fenretinide-induced Caspase 3 activity and expression of genes involved in oxidative stress in ARPE-19 cells by sterculic acid pretreatment. (**A**) Fenretinide-induced caspase 3 activity is prevented by pretreatment with sterculic acid (SA). The cultured cells were treated with 5 M fenretinide in the presence or absence of 1 h pretreatment with SA, or with C3I, a caspase 3-specific inhibitor. Caspase 3 activity was estimated as described under “Experimental Procedures”; (**B**) Fenretinide-induced HO-1 (HMOX) mRNA expression is prevented by pretreatment with SA. (**C**) Fenretinide-induced ATF3 mRNA expression is prevented by pretreatment with SCD inhibitor SA. (**D**) Fenretinide-induced GADD153 mRNA expression is prevented by pretreatment with SA. The ARPE-19 cells were treated with fenretinide in the presence or absence of SA, and the total RNA preparations were analyzed by qPCR as described under “Experimental Procedures”. The values are mean ± SD of three independent experiments performed in quadruplicate. To test for significant differences between treatment groups, one-way analysis of variance (ANOVA) was used. In the graphs, ‘*a*’ indicates statistical significance difference between fenretinide treated and untreated controls, and ‘*b*’ indicates statistical significance between fenretinide treated and fenretinide treated with inhibitors, and inhibitors alone (^*a b*^p < 0.001).

We looked at caspase-3 activation to examine whether fenretinide-induced apoptosis in ARPE-19 cells is mediated through ER stress. Caspases are cysteine proteases which normally reside in cells as inactive proenzymes and are activated by proteolytic cleavage during programmed cell death^28^. An approximately six-fold increase in the caspase-3 activity was observed in fenretinide-treated ARPE-19 cells (Fig. 2A). However, pretreatment of ARPE-19 cells with 10 μM sterculic acid effectively blocked this fenretinide-induced increase in caspase-3 activity and was similar to the inhibitory action of caspase-3 inhibitor (C3I; Ac-DEVD-CHO; Fig. 2A). We analyzed HO-1 (a cytoprotective protein that promotes and supports cell survival during oxidative stress^29,30^) expression following fenretinide treatment in the presence or absence of sterculic acid (Fig. 2B). A 70-fold increase in HO-1 expression over control was observed with 5 μM fenretinide at 24 h. This induction was effectively blocked by sterculic acid.

Mitochondrial dysfunction and ATP shortage has been shown to contribute to cellular ROS production and is also one of the various means contributing to ER stress induction^31^. As shown in Fig. 2C, fenretinide induces the expression of ATF3 (an ER stress marker involved in cellular stress responses^32^), supporting the notion that ATF3 expression contributes to apoptosis, and this was effectively blocked in cells pretreated with sterculic acid; sterculic acid alone did not induce ATF3. GADD153 (growth arrest and DNA damage-inducible protein 153; a transcription factor and another known marker for ER stress in regulating apoptosis in response to cellular stress^33,34^) mRNA expression was markedly increased in cells treated for 24 h with 5 μM fenretinide, compared to controls (Fig. 2D). In contrast, in cells pretreated with 10 μM sterculic acid, fenretinide did not induce GADD153 expression; 10 μM sterculic acid alone did not increase GADD153 expression.

### Sterculic acid pretreatment has no effect on fenretinide inhibition of ceramide synthesis in ARPE-19 cells

**Figure 3:**
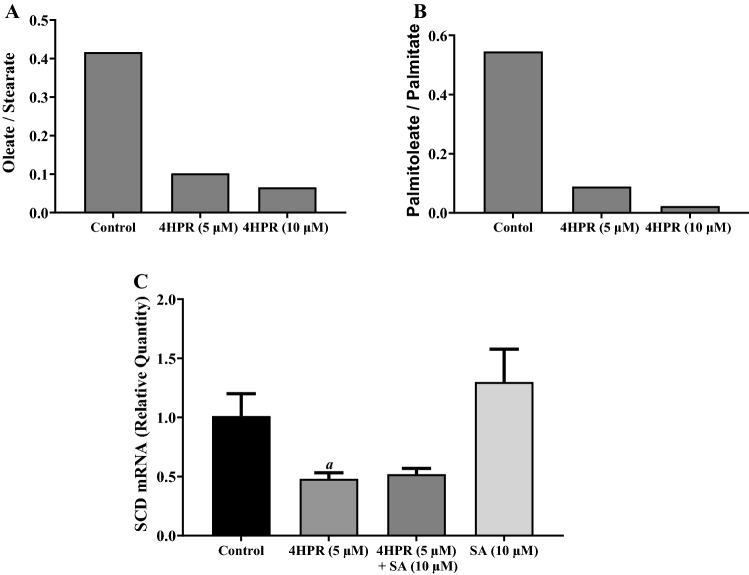
SCD enzyme activity is decreased by fenretinide in ARPE-19 cells. ARPE-19 cells were treated with or without fenretinide (4HPR) for 16 h before incubating with 50 M methyl-D3-stearate or 50 M methyl-D3-palmitate for 5 h. Lipids were extracted then analyzed by LC–MS. (**A**) The histogram shows the effect of fenretinide on the ratio of oleate to stearate. (**B**) The histogram shows the effect of fenretinide on the ratio of palmitoleate to palmitate. (**C**) Fenretinide-induced SCD mRNA expression is prevented by pretreatment of cells with SCD inhibitor sterculic acid (SA). The ARPE-19 cell cultures were treated with fenretinide in the presence or absence of SA, and the total RNA preparations were analyzed by qPCR as described under “Experimental Procedures”. The values are mean ± SD of three independent experiments performed in quadruplicate. To test for significant differences between treatment groups, one-way analysis of variance (ANOVA) was used. In the graphs, ‘*a*’ indicates statistical significance difference between fenretinide treated cells and untreated controls (^*a*^p < 0.01).

Previously, we have shown that atRA treatment increases SCD mRNA expression with a corresponding increase in its activity in ARPE-19 cells^20^. Therefore, we investigated whether fenretinide treatment modulates the expression and activity of SCD in ARPE-19 cells. We incubated both control and 16 h fenretinide-treated ARPE-19 cells with methyl-D3-stearic acid or methyl-D3-palmitic acid for 5 h and analyzed the formation of D3-oleic acid or D3-palmitoleic acid by LC–MS as an index of SCD enzyme activity. As shown in Fig. 3A,B, fenretinide treatment resulted in a concentration dependent decrease in SCD activity as shown by the decrease in the formation of D3-oleic acid and D3-palmitoleic acid. A more than 80% decrease in SCD activity over controls was observed with 10 μM fenretinide. To correlate this with SCD mRNA expression, we measured SCD mRNA expression in ARPE-19 cells treated with 5 μM fenretinide for 24 h in the presence or absence of 10 μM sterculic acid. As expected, fenretinide treatment caused SCD mRNA expression to decrease (Fig. 3C) but 10 μM sterculic acid did not reverse this; 10 μM sterculic acid alone did not alter SCD mRNA expression, as compared to control.

**Figure 4:**
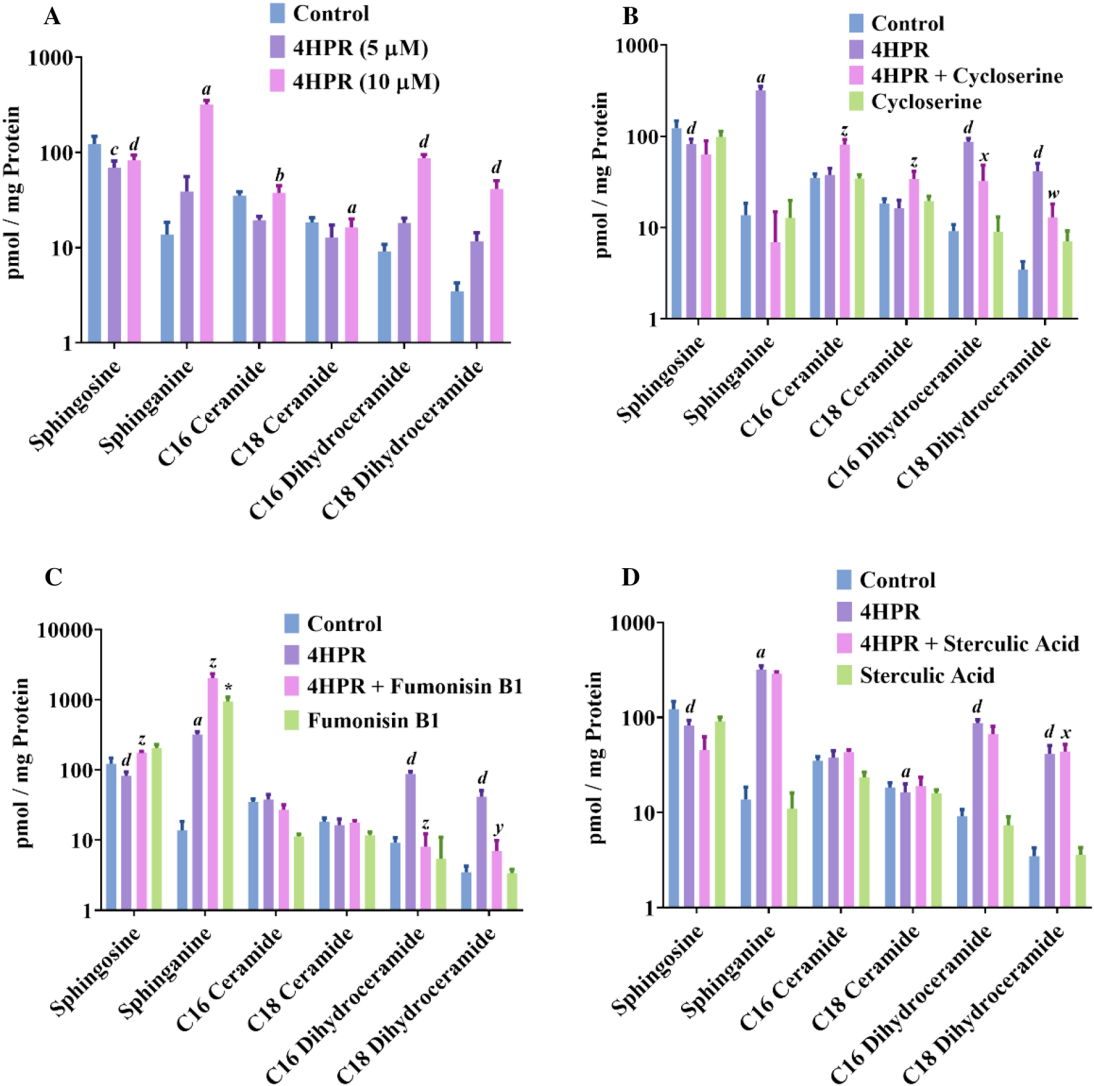
Inhibition of ceramide synthesis by fenretinide in ARPE-19 cells. ARPE-19 cells were treated with or without fenretinide for 16 h in the presence or absence of various specific inhibitors, and lipids were extracted and analyzed by LC–MS as described under “Experimental Procedures”. (**A**) the effect of fenretinide on various ceramide analogs. (**B**) the effect of l-cycloserine on fenretinide-induced ceramide synthesis. (**C**) the effect of fumonisin B1 on fenretinide-induced ceramide synthesis. (**D**) The effect of sterculic acid (SA) on fenretinide-induced ceramide synthesis. Ceramide and sphingolipid peak areas are expressed normalized to protein concentration and plotted on log scale (log_10_) due to the large differences in levels between treatment groups and untreated controls. Results are representative of 2 independent experiments analyzed in 3 or 6 repetitions and are shown as mean ± SD. To test for significant differences between treatment groups, one-way analysis of variance (ANOVA) was used. When significant treatment effects were found, the Tukey–Kramer multiple comparison test was used to determine whether there were significant differences between individual treatment groups. In the graphs, *a*,* b*,* c* and *d* indicates statistical significance difference between fenretinide treated and untreated controls (^*a*^p < 0.01; ^*b*^p < 0.05; ^*c*^p < 0.001; ^*d*^p < 0.0001), w, x, y and z indicate statistical significance difference between fenretinide treated and fenretinide treated with inhibitors (^*w*^p < 0.01; ^*x*^p < 0.05; ^*y*^p < 0.001; ^*z*^p < 0.0001), and * indicate statistical significance difference between control and Fumonisin B_1 _treated (*p < 0.0001).

Since there is compelling evidence that ceramide plays a critical role in apoptosis, proliferation, cellular senescence, and gene regulation within many cells^35,36^, we measured sphinganine, C16- and C18-ceramide and dihydroceramide levels in ARPE-19 cells after treatment for 24 h with 5 and 10 μM fenretinide (Fig. 4A). In addition, we also measured sphingosine, a metabolic product of sphingomyelins, also shown to be involved in the regulation of cell proliferation, differentiation and apoptosis^37^. The amounts of dihydroceramides and of sphinganine were increased with increasing concentration of fenretinide compared to control, but there was no significant increase in either C16- or C18-ceramide (Fig. 4A) [*p = 0.035 for C18-ceramide]. Next, ARPE-19 cells were treated with 10 μM fenretinide with or without L-cycloserine, a pharmacological inhibitor of serine palmitoyl transferase 1 (SPT1), a key enzyme in sphingolipid biosynthesis^38^. l-Cycloserine inhibited the increase in both sphinganine and dihydroceramide levels caused by treatment of ARPE-19 cells with 10 μM fenretinide for 24 h compared to controls (Fig. 4B). Conversely, there was a moderate but significant increase in C16- and C18-ceramide levels in cycloserine + fenretinide treatment compared to fenretinide treatment alone (P < 0.0001 and P < 0.0001, respectively). This might reflect a possible combined effect of cycloserine + fenretinide in increasing ceramide production from sphingomyelin degradation. There was no significant increase in C16- and C18-ceramide or dihydroceramide levels when cells were treated with L-cycloserine alone.

To further clarify whether dihydroceramide or ceramide are involved in fenretinide-induced apoptosis in human RPE cells, we pretreated cells with fumonisin B_1_, a dihydroceramide synthase inhibitor that blocks the de novo sphingolipid biosynthesis of dihydroceramide and ceramide from sphinganine^39^, prior to adding different concentration of fenretinide. We found that fumonisin B_1_ had little effect on formation of C16- and C18-ceramide and reduced C16- and C18-dihydroceramide (Fig. 4C), but increased the formation of sphinganine, indicating that both dihydroceramide and ceramides are downstream of sphinganine. In addition, fumonisin B_1_ itself increased free sphinganine formation/accumulation in ARPE-19 cells as it blocks ceramide synthase, a reflection of its in-situ inhibition of sphingolipid biosynthesis concomitant with reduction in the total mass of sphingolipids. We next examined the effect of sterculic acid on endogenous sphingolipids in cells treated with 10 μM fenretinide (Fig. 4D). We observed an increase in sphinganine and C16- and C18-dihydroceramide formation with fenretinide treatment. Sphinganine, a precursor of ceramide, has been shown to act as a negative regulator of cell proliferation and as a messenger of apoptosis^40^. Like fumonisin B_1_, sterculic acid pretreatment did not appreciably block the fenretinide-induced formation of sphinganine. On the other hand, sterculic acid alone does not affect sphinganine, ceramide or dihydroceramide levels. Taken together, these data exclude a ceramide-mediated pathway playing a major role in fenretinide-induced apoptosis in ARPE-19 cells.

### Sterculic acid pretreatment prevents fenretinide-induced ROS generation in ARPE-19 cells

**Figure 5:**
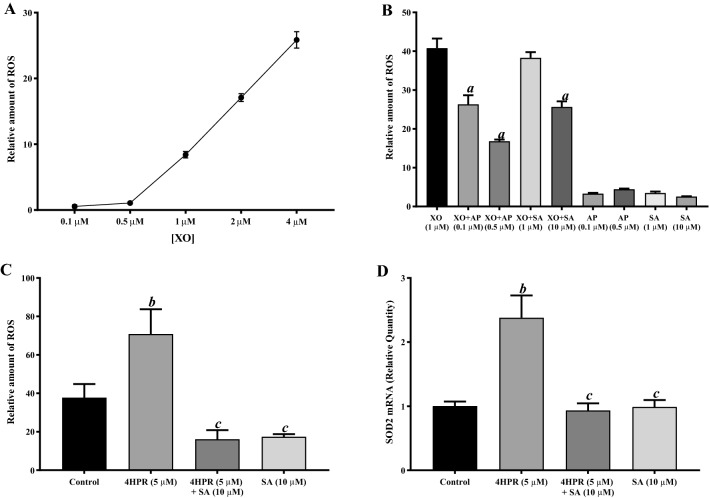
Fenretinide-induced ROS generation is blocked by pretreatment with sterculic acid in ARPE-19 cells. Fenretinide-induced ROS generation in ARPE-19 cells was measured in the presence or absence of pretreatment with sterculic acid (SA) and allopurinol using Amplex Red, as described under “Experimental Procedures”. (**A**) standard curve of superoxide formation at indicated xanthine oxidase concentrations. (**B**) inhibition of 1 μM xanthine oxidase-induced superoxide generation by SA and the ROS scavenger allopurinol. (**C**) Fenretinide-induced ROS generation in ARPE-19 cells is prevented by pretreatment with SA. (**D**) Fenretinide-induced SOD2 mRNA expression is prevented by pretreatment with SA. ARPE-19 cells were treated with 5 μM fenretinide (4HPR) for the indicated time, and total RNA preparations were analyzed by qPCR as described under “Experimental Procedures”. Intracellular ROS generation was measured using cell-permeable and nonfluorescent carboxy-H_2_DCFDA to fluorescent carboxy dichlorofluorescein (DCF) inside the cells. The values are mean ± SD of three independent experiments performed in triplicate. To test for significant differences between treatment groups, one-way analysis of variance (ANOVA) was used. In the graphs, ‘*a*’ indicates statistical significance difference between xanthine oxidase treated and xanthine oxidase treated with allopurinol or SA (^*a*^p < 0.001), ‘*b*’ indicates statistical significance difference between untreated controls and fenretinide treated (^*b*^p < 0.001), and ‘*c*’ indicates statistical significance difference between fenretinide treated and fenretinide treated + SA, or SA alone (^*c*^p < 0.001).

We next investigated the role of reactive oxygen species (ROS) in fenretinide-induced apoptosis in the presence or absence of sterculic acid (Fig. 5). To investigate a possible direct effect of sterculic acid on ROS generation, we used xanthine oxidase (XDH; present in nearly all species^41^) and a potential generator of superoxide in RPE, as a model and tested the inhibitory activity of sterculic acid towards it. Figure 5A shows a typical xanthine oxidase standard curve where the Amplex Red fluorescence values obtained relate to the amount of xanthine oxidase used. We compared sterculic acid with allopurinol, a potent inhibitor of XDH (Fig. 5B). More than 50% inhibition of XDH activity was observed with 0.5 μM allopurinol; allopurinol alone did not cause an increase in ROS generation. Similarly, ROS generation by XDH was inhibited by sterculic acid in a dose dependent manner. Cells treated with 5 μM fenretinide and assayed by carboxy-H_2_DFCDA showed more than twofold increased ROS production, relative to control values (Fig. 5C). Next, we preincubated ARPE-19 cells with 10 μM sterculic acid before treating the cells with 5 μM fenretinide for 24 h, and this completely prevented ROS production. We then examined how 5 μM fenretinide affected the mRNA expression in ARPE-19 cells of superoxide dismutase 2 (SOD2), an important enzymatic antioxidant and a first-line defense mechanism against ROS^42^, with or without sterculic acid pretreatment. Fenretinide treated cells had significantly increased SOD2 mRNA expression compared to controls, and this was markedly reduced by pretreatment with sterculic acid; sterculic acid pretreatment alone did not increase SOD2 mRNA expression (Fig. 5D).

### Sterculic acid pretreatment prevents fenretinide-mediated induction of PLA_2_ and PPARγ and activation of NF-κB

**Figure 6:**
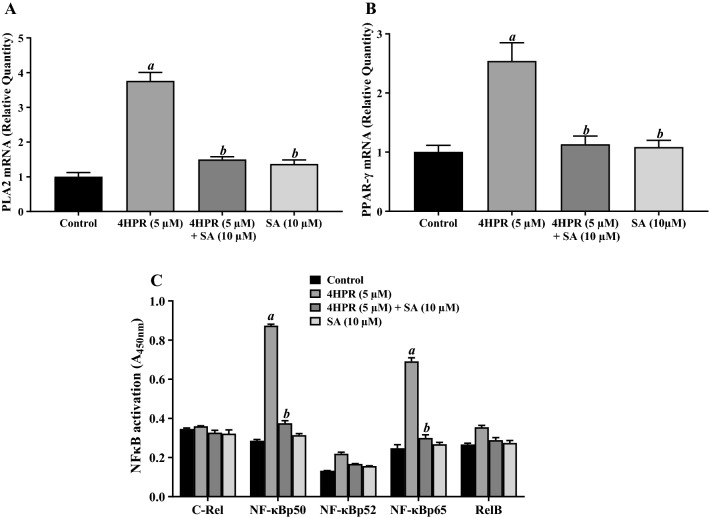
Fenretinide-induced PLA_2_ and PPARγ mRNA expression and activation of NF-κB in ARPE-19 cells are prevented by pretreatment with sterculic acid. (**A**) Fenretinide-induced PLA_2_ mRNA expression is prevented by pretreatment with sterculic acid (SA). (**B**) Fenretinide-induced PPARγ mRNA expression is prevented by pretreatment with SA. ARPE-19 cells were treated with 5 μM fenretinide (4HPR) in the presence or absence of SA, and the total RNA preparations were analyzed by qPCR as described under “Experimental Procedures”. (**C**) Fenretinide-induced NF-κB activation is prevented by pretreatment with SA. The cells were treated in the presence or absence of SA, and NF-κB activation was quantified using a TransAM NF-κB kit as described under “Experimental Procedures”. The values are mean ± SD of three independent experiments performed in duplicate. To test for significant differences between treatment groups, one-way analysis of variance (ANOVA) was used. In the graphs, ‘*a*’ indicates statistical significance difference between fenretinide treated and untreated controls, and ‘*b’* between fenretinide treated and fenretinide treated + SA, or SA alone (^*a,b*^p < 0.001).

The phospholipase A_2_ (PLA_2_) family hydrolyze membrane phospholipids and have been implicated, directly or indirectly, in cellular responses to stress by altering cell functions^43,44^. To elucidate if PLA_2_ plays a role in fenretinide-induced oxidative stress, we treated ARPE-19 cells with 5 μM fenretinide for 24 h and measured cytosolic PLA_2_ mRNA levels by RT-PCR. We found that fenretinide treatment greatly increased PLA_2_ mRNA compared to control (Fig. 6A). However, when ARPE-19 cells were pretreated with 10 μM sterculic acid for 1 h before treating with 5 μM fenretinide for 24 h, PLA_2_ mRNA expression was markedly decreased; sterculic acid alone had no effect on basal expression of PLA_2_. Peroxisome-proliferator activator receptor γ (PPARγ) has been implicated in oxidative stress response and plays a central role in quenching and containment of damage, and in fostering cell survival^45^. We found that 5 μM fenretinide treatment significantly increased PPARγ mRNA expression compared to controls, but this was significantly reduced by 10 μM sterculic acid pretreatment prior to treatment with fenretinide (Fig. 6B). Activation of the nuclear factor-kappa B (NF-κB) signaling pathway is a hallmark of biological processes in regulating numerous target genes, including apoptosis related genes^46^. ARPE-19 cells were treated with 5 μM fenretinide to determine its effect on transcriptional activity of NF-κB. As expected, NF-κB transcriptional activity was increased by fenretinide treatment (Fig. 6C). In particular, we found an increase in NF-κBp50 and NF-κBp65, the most common heterodimer of the NF-κB signaling pathway. However, sterculic acid pretreatment prevented the fenretinide-induced activation of both NF-κBp50 and NF-κBp65.

## Discussion

**Figure 7:**
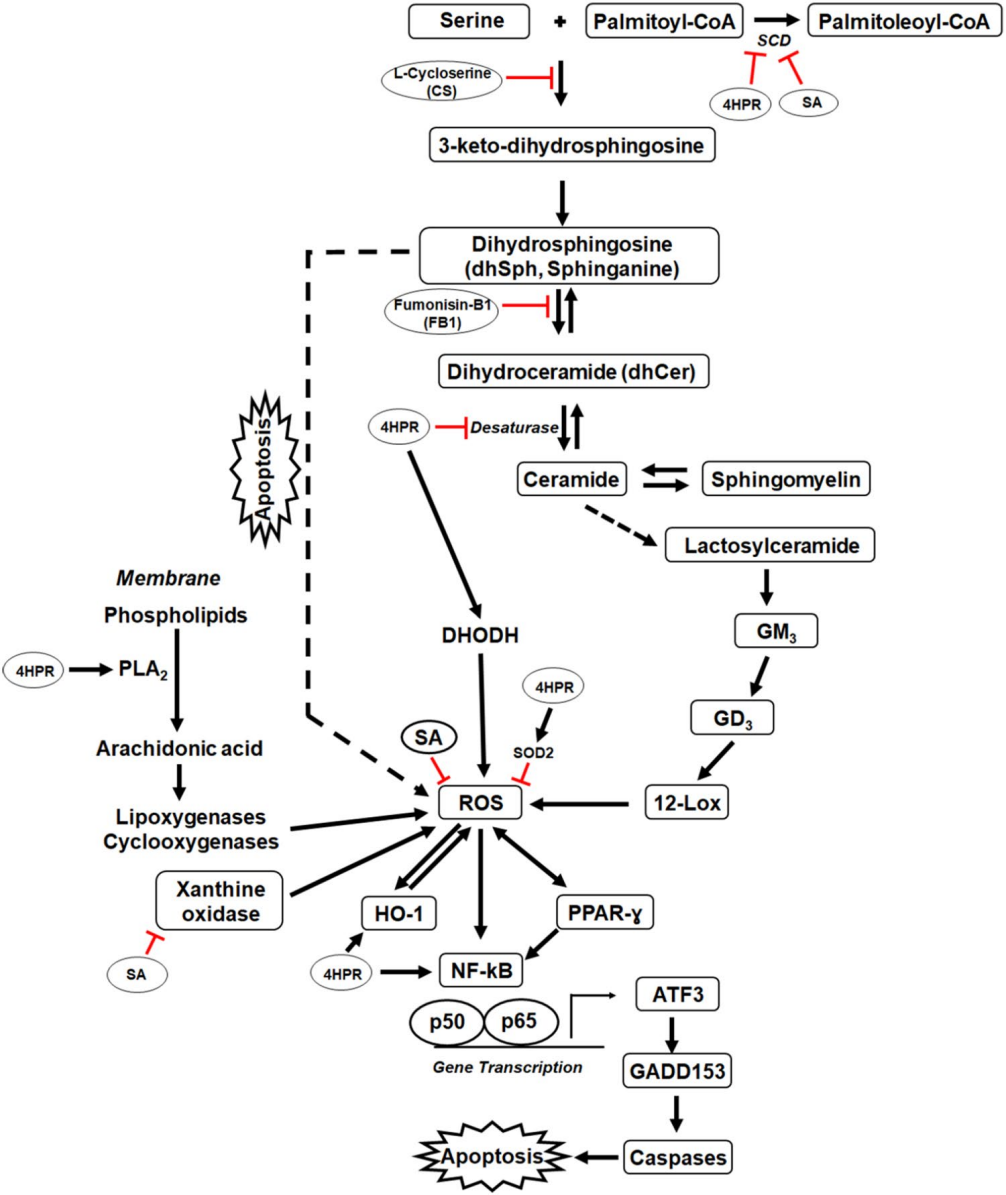
A schematic representation of modulation by sterculic acid of fenretinide-induced ROS generation and apoptosis in human RPE cells. As a naturally occurring inhibitor of SCD, sterculic acid (SA) inhibits the conversion of saturated fatty acids (palmitate, stearate, etc.) to mono-unsaturated fatty acid (palmitoleate, oleate, etc.) catalyzed by SCD. SA, if it only inhibits SCD, would be expected to be pro-apoptotic as SCD inhibition favors ceramide synthesis. However, we show here that pretreatment with SA: (i) inhibits xanthine oxidase-catalyzed ROS formation; (ii) suppresses fenretinide-induced ROS generation that leads to downregulation of the expression of downstream mediators (ATF3, GADD153 and HO-1) and activation of caspase-3; and (iii) mitigates caspase-mediated apoptosis in ARPE-19 cells. Arrows indicate putative direction of effect, and a blunted line represents an inhibitory effect. Dotted line represents a “potential” formation. *12-Lox* 12-lipoxygenase, *4HPR* Fenretinide (*N*-(4-hydroxyphenyl)-retinamide, *DHODH* dihydroorotate dehydrogenase, *GM3 and GD3* gangliosides, *HO-1* heme oxygenase 1, *PLA*_*2*_ phospholipase A_2_, *ROS* reactive oxygen species, *SA* sterculic acid, *SCD* stearoyl-CoA-desaturase.

In this study, we show that fenretinide, a synthetic amide analog of atRA, induces apoptosis in the human RPE cell line ARPE-19 by a ROS-mediated process but that pretreatment of cells with sterculic acid prevents this fenretinide-induced process. Sterculic acid pretreatment also reduced fenretinide-induced generation of ROS and other manifestations of oxidative stress. We see that the fenretinide-induced apoptosis in ARPE-19 cells is mediated by downstream NF-kB pathway signaling but is prevented by sterculic acid pretreatment. Taken together, these data suggest that sterculic acid has a potent antioxidant function modulating various oxidative pathways (Fig. 7), such as might be involved in disorders including age-related macular degeneration (AMD). However, these effects appear to be independent of sterculic acid’s effects on SCD activity.

While we find that fenretinide induces apoptosis in ARPE-19 it has also been shown to effectively block the formation of lipofuscin fluorophores such as A2E in the RPE of *Abca4* knockout mice^47^ and so has been proposed as a therapy of lipofuscin-based retinal diseases such as Stargardt disease^47^ and AMD^48^, in spite of its known toxicity and use as a chemotherapeutic ^22–25^. Significantly, we^49^ have found that physiological metabolites of fenretinide might underlie its pleiotropic nature in interfering with retinal binding/transport, cell survival, inducing apoptosis, and in insulin and glucose homeostasis^23,26,50–52^. However, we found that pretreatment with sterculic acid effectively forestalls fenretinide-induced apoptosis in ARPE-19 cells and here we examine the possible mechanism of this effect.

Sterculic acid is a naturally occurring inhibitor of SCD activity. SCD is important for regulation of the unsaturated fatty acid to saturated fatty acid ratio that is thought to control bilayer fluidity and membrane function^53^. Increased saturated fatty acid concentration reduces membrane fluidity resulting in impaired cellular functions and/or cell death^54^. Increased SCD expression is seen in several types of cancer and protects these from the toxic effects of saturated fatty acids^55^. While decreased SCD activity reduces proliferation of cells and make them more sensitive to apoptosis^56^, SCD inhibitors reduced xenograft colorectal tumor growth and induced apoptosis^15,57^. In this regard, sterculic acid reduced proliferation, increased apoptosis, and reduced desaturation ratio in prostate cancer cells^19^. In our study, we observed that sterculic acid pretreatment inhibited fenretinide-induced apoptosis and changes in cell morphology in ARPE-19 cells, correlating with the notion that sterculic acid induces cellular effects independent of its effects on SCD activity^58^.

We showed earlier that fenretinide-induced ARPE-19 cell death occurs via caspase activation and activation of ER stress markers mediated through RARs^23^. Here we confirm these findings, showing activation of caspase-3^59^, increase in ATF3^60,61^ mRNA expression, induction of HO-1^23,49,62^, and of GADD153^33,34^ in response to fenretinide treatment, correlating with responses to stress such as ROS. Importantly, sterculic acid pretreatment prevents these fenretinide-induced increases, indicating its strong antioxidant potential. Consistent with our results, sterculic acid also inhibits 7-ketocholesterol-mediated induction of ATF3 and GADD153 mRNA in ARPE-19 cells^63^.

Ceramides are important second messengers that impinge on several pathways such as oxidative stress, inflammation and apoptosis^64^. In our experiments, we observed an increase in sphinganine levels after 24 h of fenretinide treatment. Increase in sphinganine has been shown to mediate fenretinide-induced apoptosis, whereas sphingosine, which changed little, is mitogenic and inhibits apoptosis^65,66^. The observed increase in dihydroceramide formation, but not of ceramide, following fenretinide treatment agrees with earlier findings that oxidative stress and dihydroceramide accumulation are early and distinct events in fenretinide-induced cell death^65,67,68^. Further, use of l-cycloserine, an inhibitor of SPT1, and of fumonisin B_1_, an inhibitor of ceramide synthase, implicates an effect on upstream ceramide metabolism rather than on sphingomyelinase activation^69,70^. Also, the lack of effect of sterculic acid pretreatment on fenretinide-induced dihydroceramide accumulation suggests that an antioxidant mechanism may be preventing fenretinide-mediated apoptosis. Earlier, we found that ROS generation mediated fenretinide-induced apoptosis in ARPE-19 cells ^23^, so sterculic acid inhibition would be antioxidant/anti-apoptotic in nature.

ROS are essential at low to modest levels to regulate normal physiological functions such as cell proliferation, differentiation, cell death, and play an important role in the maintenance of redox balance^71–73^. On the other hand, enhanced ROS production is associated with numerous pathologies including AMD and Alzheimer diseases^73,74^. We found that sterculic acid can inhibit xanthine oxidase-induced ROS generation comparably to allopurinol^75^, and that it also blocks fenretinide-induced ROS generation in ARPE-19 cells. In addition, we find that fenretinide-induced SOD2 mRNA expression in ARPE-19 cells is blocked by sterculic acid. SOD2 catalyzes the removal of superoxide species; SOD mimetics are used to inhibit oxidative stress-induced pathologies^76^. This suggests that ROS generation plays a major role in fenretinide-induced apoptosis in RPE cells, and that sterculic acid pretreatment can prevent this ROS generation.

In addition, sterculic acid pretreatment prevents fenretinide-induced increase in PLA_2_ mRNA expression_._ This would turn off arachidonic acid (AA) synthesis and downstream oxidative stress mediators and lysophospholipids, involved in cell proliferation, apoptosis, alteration of mitochondrial function, etc.^77,78^. As blocking AA-derived metabolites ameliorates tissue damage in different CNS insults^79^, development of further PLA_2_ inhibitors will be important^80^. The PPARs are involved in limiting physiological and pathological processes involving ROS^81,82^, as they are activated by fatty acids and fatty-acid derived eicosanoids^83,84^. Here, we show that fenretinide induces the expression of PPARγ mRNA in ARPE-19 cells, and that pretreatment with sterculic acid prevents this. Consistent with our results, it has been shown that fenretinide also acts as a PPARγ ligand to increase its activity^85^. In addition, inhibition of PPARγ reversed fenretinide’s pro-inflammatory effects further indicating that fenretinide is a PPARγ ligand^86^, and implicating PPARγ in fenretinide-induced apoptosis in ARPE-19 cells. Furthermore, pretreatment with sterculic acid reduces many genes implicated in cell death and PPAR-mediated pathways, etc.^87^.

Species p50 and p65 comprise the most common nuclear factor-κB (NF-κB) signaling pathway heterodimer involved in multiple physiological and pathological processes^88^. We observed that downregulation of p50/p65 activation is associated with prevention of fenretinide-induced apoptosis of ARPE-19 cells by pretreatment with sterculic acid. This indicates that fenretinide-induced apoptosis in ARPE-19 cells is mediated through downstream NF-κB activation and correlates well with the pro-apoptotic role of NF-κB in neuronal cells, and its activation as a hallmark of ER stress^89^. Consistent with our results, fenretinide-induced apoptosis in SH-SY5Y neuroblastoma cells is mediated via ROS generation activating the NF-κB pathway^90^.

In summary, we present data demonstrating that the cyclopropenoic fatty acid sterculic acid prevents fenretinide-induced NF-κB-mediated apoptosis in human ARPE-19 cells, and that its inhibitory action is mediated through an antioxidant effect and is independent of its effects on SCD activity. Thus, we demonstrate the potential beneficial function of sterculic as an antioxidant that can forestall apoptosis. This will further stimulate efforts to determine a therapeutic role for sterculic acid in treating retinal and other diseases.

## Methods

### Cell culture

The human adult RPE cell line-19 (ARPE-19) was obtained from ATCC at passage 18, and the line used in these experiments was validated by the ATCC Cell Line Authentication Service (Promega, Madison, WI) using tandem repeat analysis plus the Amelogenin gender determining locus and was a perfect match for the ATCC human cell line CRL-2302 (ARPE-19). The cells were grown in Dulbecco’s modified Eagle’s medium (DMEM) containing nutrient mixture F12 (Cellgro, Herndon, VA) supplemented with 5% fetal bovine serum, penicillin (100 U/ml) and streptomycin (100 μg/ml) as described previously^20^. In all the studies reported here, ARPE-19 cells at passages 18 to 22 were used. Cells were seeded onto 6-well tissue culture plates at a density of 2 × 10^5^ cells/ml in complete medium and allowed to grow overnight. Fenretinide was added to the culture medium when the cells become 75 to 85% confluent and the cells were allowed to grow for additional indicated time intervals. Sterculic acid and 9-thia stearate (9-Thia)^91^ were added 1 h prior to the addition of fenretinide. All compounds were dissolved at a concentration of 10 mM in DMSO before adding to the cell culture medium. The controls received the same amount or 0.1% of DMSO. The cells were maintained at 37 ˚C in a humidified environment of 5% CO_2_ in air.

### Apoptosis ELISA

Detection of apoptosis in ARPE-19 cells was performed by quantitative sandwich-enzyme-immunoassay using mouse monoclonal antibodies directed against DNA and histones (Cell Death Detection ELISA kit, Roche). Briefly, the cells were seeded at a density of 2 × 10^4^ cells/well in a 24-well tissue culture plate. After 24 h, the cells were treated with or without fenretinide in the presence or absence of 9-thia stearate and sterculic acid and were allowed to grow for additional 24 h. The cells were lysed by adding lysis buffer (250 μl/well) and incubated for 30 min at room temperature. The cell lysates were then centrifuged at 250×*g* for 10 min, and 10 μl of supernatants was removed and analyzed by ELISA, quantitated by measuring the absorbance at 405 nm using a Victor2 Multilabel Counter (Perkin Elmer).

### Quantitative Real-Time RT-PCR

For quantitative real-time RT-PCR (qPCR), 2 μg total RNA extracted from ARPE-19 cells with RNeasy Protect Mini Kit (Qiagen) was reverse transcribed using High-Capacity cDNA Archive Kit^92^ (Applied Biosystems). After reverse transcription, 5 μl cDNA was used as templates for qPCR performed on an Applied Biosystems 7500 Real-Time PCR System using TaqMan Universal PCR Master Mix and other reagents from Applied Biosystems following manufacturer’s default thermal cycling conditions. Each PCR reaction was set up in 20 μl triplicates using validated TaqMan probes and primers of GADD153 (assay identification number Hs00358796_g1), HO-1 (Hs00157965_m1), ATF3 (Hs00231069_m1), SOD2 (Hs00167309_m1), PPAR-γ (Hs0111513_m1), PLA2 (Hs00179898_m1), and SCD (Hs00167309_m1). Human GAPDH gene (catalog number 4326317E) was used as endogenous control. The gene specific probes were labeled with reporter dye FAM, and the endogenous control GAPDH was 5′-labeled with a different reporter dye, VIC. Gene amplification data were analyzed with an Applied Biosystems 7500 System Sequence Detection Software version 1.2.3. The results were expressed as *n*-fold induction or inhibition in gene expression relative to endogenous control calculated using the ΔΔC_T_ method.

### Reactive oxygen species (ROS) measurement

Intracellular generation of ROS was measured with carboxy-H_2_DCFDA, cell-permeable and nonfluorescent when applied to cells and oxidized by ROS to fluorescent carboxy dichlorofluorescein (DCF) inside the cells. Briefly, cells seeded in 6-well plates (2 × 10^4^ cells/well) and treated with or without fenretinide were incubated with 5 μM carboxy-H_2_DCFDA for 15 min at 37 °C. In some assays, cells were incubated for 1 h with antioxidants or sterculic acid prior to the addition of the dye and treatment with fenretinide. Then the cells were washed twice with 0.01 M phosphate-buffered saline (PBS), trypsinized, and resuspended in OptiMem® I medium (Invitrogen). The resulting fluorescence was measured using a Victor2 Multilabel Counter using an excitation wavelength of 480 nm and an emission wavelength of 530 nm.

### Caspase activity assays

The activity of caspase proteases was measured using an ApoAlert Caspase Profiling kit (BD Biosciences). Briefly, whole cell lysates from treated samples were added to the wells of a 96-well plate containing immobilized caspase-3-specfic substrates covalently linked to the fluorogenic dye 7-amino-4-methyl coumarin (AMC) and incubated for 2 h at 37 °C. Cell lysates pre-incubated with 5 μM caspase 3-specific inhibitor caspase 3-I (C3I; Ac-DEVD-CHO) were also added to wells containing immobilized caspase-specific substrates. Fluorescence was measured using a Victor2 Multilabel Counter with excitation and emission wavelengths of 380 nm and 460 nm, respectively.

### Lactate dehydrogenase (LDH) release

LDH assays were performed in the culture supernatant using Cytotoxicity Detection kit ^27^ (Roche). Cell-free culture supernatants of treated and control samples were incubated with 1 mM pyruvate and 0.2 mM NADH in 0.1 M Tris–HCl (pH 7.4) in a volume of 1 ml followed by the addition of tetrazolium salt. The amount of formazan dye formed in the supernatant was measured at 490 nm using a Victor2 Multilabel Counter.

### Xanthine oxidase (XDH) assay

The XDH assays were performed in vitro using the Amplex^®^ Red Xanthine/Xanthine Oxidase Assay Kit (Thermo Scientific) in which superoxide generated by xanthine oxidase (XDH) activity reacts stoichiometrically with Amplex Red reagent to generate resorufin, a red-fluorescent oxidation product. XDH activity was measured by adding 50 μM Amplex Red, 0.2 U/ml horseradish peroxidase (HRP), 0.1 mM hypoxanthine and the indicated amount of XDH. Allopurinol (AP; 0.1 and 0.5 μM) and sterculic acid (SA, 1 and 10 μM) were added to the reaction mixture with or without XDH to study their inhibitory functions. Reaction buffer without XDH used as a negative control. After 30 min, the generation of fluorescent resorufin was measured using a Victor2 Multilabel Counter with absorption and fluorescence emission maxima of 571 nm and 585 nm, respectively. A background of 65 fluorescence units was subtracted from each data point. Assays were done in triplicate.

### NF-κB transcription factor activity assay (NF-κB-assay)

The activity of individual NF-κB subunits (p50, p52, p65, c-Rel, and RelB) was detected by an ELISA-based NF-κB family transcription factor assay kit (Active Motif, Carlsbad, CA, USA). Briefly, nuclear extracts (2 μg) prepared from ARPE-19 cells treated with fenretinide in the presence or absence of sterculic acid were incubated in 96-well plates, and immobilized with consensus double stranded oligonucleotides of NF-κB binding sequence (5′-GGGACTTTCC-3′), for 1 h at RT. The captured complexes were incubated with individual NF-κB antibodies (1:1000) for 1 h, and subsequently with HRP conjugated secondary antibody (1:1000) for 1 h. After washing away unbound antibody the optical density (OD) value was measured at 450 nm using a Victor2 Multilabel Counter.

### Sphingolipid analysis

Cells were treated with fenretinide (5 or 10 μM), sterculic acid (10 μM), cycloserine (10 μM), fumonisin B_1_ (10 μM) or vehicle (control) for 16 h at which time cells were harvested. Total lipids were extracted into methyl-tert-butyl-ether (MTBE) using the method of Matyash et al.^93^. Extracted lipids were subjected to liquid chromatography-mass spectrometry (LC–MS) to determine the sphingolipid content. LC–MS analysis was performed using an Alliance 2695 Separation Module (Waters Corp., Milford, MA) coupled to an API 2000 triple quadruple mass spectrometer (SCIEX). Sphingolipids were resolved on a Pursuit Diphenyl reversed-phase column (2.0 × 50 mm, 3 μm; Agilent) using a 3 min linear gradient from 30 to 95% mobile phase B beginning 1 min after injection at a flow rate of 0.8 ml/min. (mobile phase A: 25 mM ammonium acetate/0.1% formic acid; mobile phase B: acetonitrile/0.1% formic acid). Mass spectral analysis of the sphingolipid species was performed using electrospray ionization in the positive ion mode with multiple reaction monitoring (MRM). The following compound-specific transitions for precursor and characteristic product ions were used: sphingosine, 300 → 264; sphinganine, 302 → 266; C16 Ceramide, 538 → 264; C16 DHCeramide, 540 → 266; C18 ceramide, 566 → 264; and C18 DHCeramide, 568 → 266. Data was acquired and analyzed using Analyst software v1.5 (SCIEX).

### Stearoyl-CoA desaturase activity

For experiments measuring SCD activity ^26^, cells were treated with either 5 or 10 μM fenretinide. After 16 h, cells were incubated with 50 μM of either methyl-D3 palmitic or stearic acid for an additional 5 h before harvesting. Cell pellets washed with PBS were resuspended in 450 μl cell lysis buffer (Cell Signaling Technology), and then sonicated on ice until uniformly dispersed. An aliquot (50 μl) of the dispersed cell suspension was used for protein quantitation, and the remaining 400 μl was used for lipid extraction as follows: samples were spiked with a small volume of pentadecanoic acid (in CHCl_3_) as an internal standard and then were subjected to saponification for 15 min at 72 °C by addition of an equal volume of 0.6 M methanolic KOH. Following saponification, samples were acidified by addition of 100 μl formic acid. The acidified mixture was extracted twice with 1 ml chloroform to recover free fatty acids. The chloroform extracts were pooled, solvent evaporated under argon at 37 °C, and the residue resolubilized in 100 μl chloroform/methanol (1:4).

Lipid extracts were analyzed by LC–MS using a modification of the procedures of Dillon et al.^94^. The LC–MS system consisted of a 1200 Series Capillary LC (Agilent, Santa Clara, CA) coupled to a Q-Tof micro mass spectrometer (Waters Corp., Milford, MA) equipped with an electrospray ionization source. Separation of fatty acids was achieved by isocratic elution on a PLRP-S polymeric reversed- phase column (2.1 × 150 mm, 3 μm; Agilent) heated to 65 °C. A mobile phase consisting of 25% acetonitrile: chloroform (1:1), 40% methanol, 25% water, 10% of 10 mM ammonium acetate was run at a flow rate of 0.1 ml/min. The mass spectral analysis of the fatty acids was performed in the negative ion mode and data were then analyzed using MassLynx software v4.1 (Waters) by extracting the ion chromatograms of the labeled and non-labeled stearic, palmitic, oleic, and palmitoleic acid peaks and measuring peak areas.

### Statistical analysis

All assays were performed with at least three repeated experiments, unless otherwise specified, expressed as mean ± standard deviation (SD), and the statistical analyses are detailed for each individual analysis in the appropriate figure caption. Statistical significance was determined by unpaired Student’s *t*-test or one-way ANOVA (analysis of variance) followed by Tukey–Kramer multiple comparison test. The alpha level for significance was p ≤ 0.05.

## Data Availability

The datasets used and/or analyzed during the current study are available from the corresponding author on reasonable request.
